# A New Approach to Assess Lifetime Dietary Patterns Finds Lower Consumption of Animal Foods with Aging in a Longitudinal Analysis of a Health-Oriented Adventist Population

**DOI:** 10.3390/nu9101118

**Published:** 2017-10-13

**Authors:** Marcia C. T. Martins, Karen Jaceldo-Siegl, Michael Orlich, Jing Fan, Andrew Mashchak, Gary E. Fraser

**Affiliations:** 1 Department of Nutrition, Adventist University of São Paulo—UNASP/SP, Estrada de Itapecerica 5859, Jardim IAE, São Paulo 05858-001, Brazil; marciactm@yahoo.com.br; 2 School of Public Health, Loma Linda University, Loma Linda, CA 92350, USA; kjaceldo@llu.edu (K.J.-S.); morlich@llu.edu (M.O.); jing_fan@yahoo.com (J.F.); mashchak@llu.edu (A.M.)

**Keywords:** diet, vegetarian, dietary patterns, Adventists, dietary recall, life-course nutrition, foods of animal origin, lifetime diet patterns

## Abstract

Life-course diet patterns may impact risk of disease, but little is known about dietary trends with aging. In a retrospective longitudinal analysis we estimated lifetime intake of animal products and adherence to vegetarian dietary patterns among 51,082 Adventist Health Study-2 (AHS-2) subjects using data from a reliable life-course dietary (meats, dairy, eggs) questionnaire. Results showed a marked tendency to consume fewer animal products (in total) in older years and to reduce consumption of meat, poultry and fish, but not eggs or dairy. Among the 29% of elderly subjects who during their lifetime kept the same dietary pattern (LTS) were: LTS-vegans (1.1%), LTS-lacto-ovo vegetarians (31.2%), LTS-pesco vegetarians (0.49%), LTS-semi vegetarians (3.7%), and LTS-non-vegetarians (63.5%). Among the 71% of switchers were “Converters” (59.7%) who moved towards and “Reverters” (9.1%) who moved away from vegetarian diets, and Multiverters (31.2%), who had moved in both directions. LTS-non-vegetarians, and also reverters, were more overweight and showed a less healthy lifestyle than others. We conclude that the dietary patterns are dynamic with strong trends to reduce animal foods and to adopt more vegetarian patterns with aging. The disease experience of subjects with different lifetime dietary patterns can be compared.

## 1. Introduction

Particularly since Belloc and Breslow (1972) drew attention to health-related lifestyle practices, a growing body of research has examined the effects of different combinations of such behaviors on health and disease outcomes. Generally, studies have shown that subjects who maximize a number of healthy behaviors will reduce their risk of chronic disease [1,2]. Thus, many common disorders are known as lifestyle-related diseases (e.g., obesity, type 2 diabetes, hyperlipidemia, hypertension, cardiovascular diseases and cancer) [3,4,5,6]. The relevant lifestyle behaviors associated with these diseases often emerge, and may begin to act early in life then continue to have impact throughout the life course. Indeed they may even affect the health of the next generation through transmitted changes in gene expression [7,8]. The three most significant broad categories of risk factors operating during the life course are smoking, physical inactivity and poor-quality diet [9].

Dietary pattern analysis is a useful approach to studying the relationships between diet and disease as it examines the effects of the overall diet instead of focusing on individual foods or nutrients. Thus, it presents a broad overview of food and nutrient consumption in simple categories [10,11]. Vegetarian dietary patterns have been associated with favorable values of a wide variety of risk factors and reduced risk of many diseases [12,13]. These include weight gain or obesity [14,15], metabolic syndrome [16], type 2 diabetes mellitus [6], hypertension [17], cardiovascular disease [18], colorectal [19], prostate [20] and overall cancers [21].

Much of the present understanding of the health effects of vegetarian diets has come from studies in which the classification of vegetarian dietary patterns is based on the degree of avoidance of animal foods (mainly red meat, poultry, fish, dairy and eggs) [22,23]. In these studies, generally favorable outcomes that have been associated with vegetarian diets have usually been based on recent intake of these foods as derived from food frequency questionnaires. However, dietary intake fluctuates during the lifetime, suggesting the need to take a life-course approach when associating diet with subsequent health experience. In particular, there is suggestive evidence that consumption earlier in life may influence later disease risk [24,25]. The possible influence of fluctuating dietary patterns over the lifetime needs further exploration.

We have previously developed a reliable, inexpensive instrument to obtain individual historical dietary habits information from the first up through the 8th decade of life. This lifelong dietary habits questionnaire requires each participant to recall the intake of five foods (red meat, poultry, fish, eggs and dairy) which are used to determine dietary patterns [26]. Thus, it allows us to classify subjects in the Adventist Health Study-2 (AHS-2) cohort according to their vegetarian dietary pattern across all decades of life [27]. This approach provides the opportunity to identify individuals who have followed the same dietary pattern for their whole life, as well as those whose diets varied during the life course, as these are potentially relevant exposures [25]. Using this tool in a retrospective longitudinal analysis, the present paper describes time trends and lifetime consumption of animal products and adherence to vegetarian dietary patterns among subjects in the Adventist Health Study-2 (AHS-2) cohort.

## 2. Materials and Methods

**Study population.** The AHS-2 is a prospective study of 96,335 members of the Seventh-Day Adventist Church in the U.S. and Canada. The design of the AHS-2 has been described in detail elsewhere [28]. Briefly, adult men and women of diverse ethnicity (mostly Caucasian or Black/African-American) and aged ≥ 30 years were enrolled between 2002 and 2007. Participants completed a baseline questionnaire that included sections on diet, demographics, physical activity, height, weight and other lifestyle practices. During follow-up after the baseline questionnaire, biennial Hospitalization History Form questionnaires (HHF) were administered. The third of these (HHF-3) gathered the pertinent dietary information from each previous decade of life, in addition to other selected non-dietary data.

**Ethics.** This study was conducted according to the guidelines laid down in the Declaration of Helsinki and all procedures involving human subjects were approved by the Loma Linda University Institutional Review Board. Written informed consent was obtained from all subjects.

**Assessment of demographic and lifestyle exposures.** For these analyses selected demographic and lifestyle variables were obtained from the AHS-2 baseline questionnaire: gender (male or female), educational level (up to high school, some college and college or higher), smoking (current, past smoking at any amount or never), current alcohol consumption of any amount (yes/no), BMI (<18.5, 18.5–24.9, 25–29.9, or ≥30.0, calculated as weight in kilograms divided by height in meters squared); also age at the third biennial questionnaire (HHF-3). Those self-identifying at least in part as Black/African, American, West Indian/Caribbean, African American, or Other Black were categorized as black and all others as nonblack. Exercise was evaluated as the time usually spent in vigorous activities, such as brisk walking, jogging, bicycling, etc. long enough or with enough intensity to “work up a sweat”, “get your heart thumping”, or to “become out or breath”. The responses were categorized as 0, 1–20, 21–60, 61–150 and ≥151 min/week. Participants also reported the average number of hours of sleep and hours per day of television watching. Responses were divided into three categories for hours of sleep: ≤6, 7, ≥8 h of sleep/day, and for TV watching: <1, 1–2, and ≥3 h/day. Years of church membership were calculated by subtracting age of baptism from current age. Participants were also classified dichotomously to lifetime Adventists if they were Adventists at entry to the study, at ages between 15–25 years, and that also either the mother or father who raised him/her was Adventist when the participant was 0–15 years of age.

**Instrument to recall vegetarian dietary patterns during the lifetime.** The third Hospitalization History Form (HHF-3) was mailed starting January 2009 through May 2010, and included items asking about the frequency of consumption of the five food groups (red meat, poultry, fish, eggs and dairy) we need to classify subjects according to a vegetarian dietary pattern. Data was gathered pertaining to decade ages (10, 20, 30, etc. years of age) up till that preceding the subject’s present age or a maximum age of 80 years. This instrument has been described elsewhere with regard to its reliability when recalling past consumption of the five animal food groups [26] and also when recalling vegetarian dietary patterns across long periods of life [27]. In particular, reliability of recall over 33 years was good for the meats (correlation coefficient *r* = 0.71, red meat; 0.67, poultry; 0.60, fish), and also for agreement with vegetarian dietary pattern (*r* = 0.72).

Among the AHS-2 participants, 63,919 completed HHF-3, on average 5.3 years after enrollment (range: 1.1–8.9 years). We excluded 20% of participants, those who had missing responses on two or more entire columns (particular foods) or rows (age decades) of the lifelong dietary habits instrument, thus finally including 51,082 in the analytical population. Those included were younger (61.9, standard deviation (SD) 12.9 versus 67.3, SD 12.9 years), less likely to be female (64.0% versus 66.1%), less likely to be black (16.4% versus 27.3%), and had attained a higher educational level (44.9% college graduates versus 32.4%) than subjects excluded. Those missing just one item included one entire row (15%) corresponding to one decade of life, or one entire column (2%), corresponding to one food category. The 1534 instances of these single missing responses were filled by multiple imputation [29], conditional on the variables age, race, gender and education, thus providing unbiased results, conditional on the missing at random assumption.

**Classification of lifetime vegetarian dietary patterns.** Dietary patterns in AHS are well characterized [22] with definitions based on the intake of five animal food groups (red meat, poultry, fish, eggs, and dairy foods) as obtained from a validated food frequency questionnaire (FFQ) [30,31]. First, three composite variables are created by calculating reported intake frequencies of: (1) meat (red meat + poultry); (2) dairy/eggs (dairy + eggs); and (3) fish as a separate group. Thus, vegans are participants who reported consuming each of meat, fish, and dairy <1 time/month. Lacto-ovo vegetarians consumed dairy ≥1 time/month, but fish and meat each <1 time/month. Pesco vegetarians consumed fish ≥1 time/month but other meats <1 time/month. Semi vegetarians are defined as consuming non-fish meats ≥1 time/month and the sum of meat and fish ≥1 time/month but ≤1 time/week. Non-vegetarians consumed non-fish meats ≥1 time/month and all meats combined (fish included) >1 time/week. There were no requirements relative to dairy or egg intake for pesco, semi and non-vegetarians. We applied these definitions to the lifetime data gathered in HHF-3 in order to classify subjects according to vegetarian dietary pattern for each decade of life as previously described by Teixeira-Martins et al. [27].

Individuals whose dietary pattern was consistent in each decade of life were labeled as “lifetime stable” (LTS) LTS-vegans, LTS-lacto-ovo vegetarians, LTS-pesco vegetarians, LTS-semi vegetarians, or LTS-non-vegetarians. The remaining participants, who changed their patterns one or more times during life, were labeled as switchers (SW). Adapting the concept described by Rosell et al. (2006) [15], subjects who, during their lifetime up to current age (at HHF-3), changed their diet in one or more steps in the direction non-vegetarian→semi vegetarian→pesco vegetarian→lacto-ovo vegetarian→vegan, were named “Converters”; conversely, subjects who changed their diet in one or more steps in the opposite direction were named “Reverters”. Subjects who during the same period changed their diet both ways were named “Multiverters”.

**Changes in consumption of animal food groups over the lifetime.** To determine changes in consumption of animal products over the lifetime, we measured the direction of changes from one decade to the following decade (e.g., between ages 10 and 20 years, 20 and 30 years, so forth, up to between 70 and 80 years). Three types of changes were possible: increase, decrease or no change. For example, if an individual reported consuming red meat 1+ per week at 30 years, and then 1–3 per month at 40 years, this would represent a decrease. The proportion of subjects who chose each option was calculated for every animal food at each change of decade.

**Duration of adherence to current dietary pattern.** We used the participant’s current vegetarian dietary pattern taken from the baseline study questionnaire to represent his/her dietary pattern in the last reported decade of life. To calculate duration of adherence to the current vegetarian dietary pattern, we summed the number of contiguous decades (including the relevant proportion of their current decade) for which the participants had the same dietary pattern as that reported for their current decade.

**Statistical methods.** Analyses were performed using SAS, version 9.3 (SAS Institute Inc., Cary, NC, USA). An alpha level of 0.05 was used to define statistical significance. Proportions of individuals in different dietary patterns at each age decade were estimated using a separate nominal multinomial regression for each age decade while adjusting for birth cohort. The probability of each dietary pattern was standardized to birth cohort at a current age of 60 years, although the model was of course informed by data from other birth cohorts. Subjects identified as Lifetime Stable or Switcher were compared according to selected demographic and lifestyle variables. Analyses of variance (ANOVAs) were used to check for differences in the means of continuous covariates and Chi-Square tests were used to check for differences in proportions among categorical covariates. Covariates used for adjustment were set at the mean levels for that analytic population when reporting results.

## 3. Results

**Figure 1:**
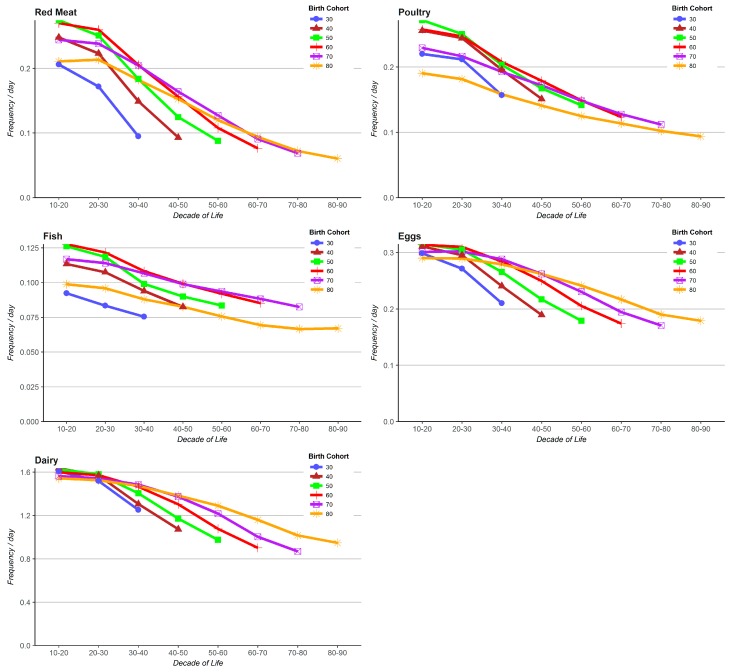
Consumption of animal products at particular ages during the observed lifetimes for different birth cohorts of the Adventist Health Study-2 (*N* = 51.082). Adjusted for gender and race.

**Estimates of lifetime consumption of animal products.** Over the lifetime the most commonly consumed animal derived food was dairy followed by eggs, poultry, red meat, and then fish (results adjusted for gender and race). In general, all birth cohorts tended to substantially reduce their daily frequency of consumption of animal products over the lifetime (Figure 1). The youngest birth cohort tended to eat less of the meats during childhood and adolescence than other cohorts, with the 50–60 year old cohort being the highest consumers across the lifespan. When they were younger (the 1930’s) the two oldest cohorts generally consumed lower amounts of all meats similar to the youngest cohort at the same ages, but then (the oldest cohort particularly) showed lower slopes of decline with subsequent ageing. The figures depicting consumption of dairy and eggs both showed that consumption of these foods during childhood and adolescence was very comparable across birth cohorts. However, each successive cohort had a more rapid decline in intake than that preceding it. Consequently at older ages the older birth cohorts were eating more of these foods at the same ages than the younger birth cohorts.

**Figure 2:**
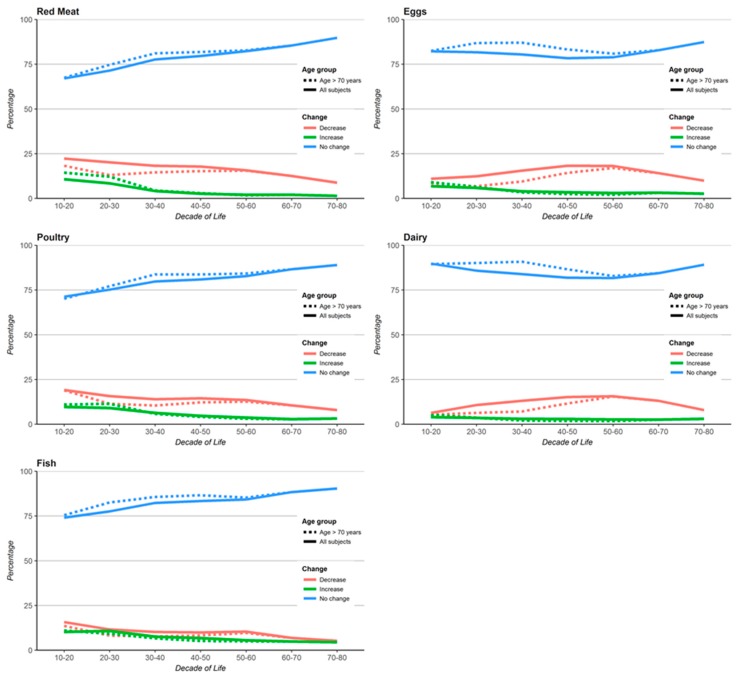
Distribution of changes (increase, decrease, no change) of consumption of animal products from one decade of life to the following decade. Dotted lines represent a sub-cohort of the oldest individuals with age ≥ 70 years (*N* = 15,332). Adjusted for gender and race.

**Patterns of change in the consumption of animal products.** The proportions of subjects who either did not change, or decreased, or increased consumption of the animal foods from one decade to the next were also evaluated for the whole cohort (adjusted for differences in gender and race). There appeared to be little difference by birth cohort as shown in the comparison for instance with the oldest birth cohort (individuals with current ages 70 years or older) (Figure 2). The proportions of subjects decreasing intakes (red) were nearly always higher than the proportions increasing intakes (green) at all ages. For red meat, poultry and fish changes were most likely during ages 20–40 years, which typically coincides with the period of adult baptism into the Adventist church. The proportion of those who did not change intake (blue) of red meat, poultry or fish increased almost linearly with age. Older subjects were more resistant to change. For eggs and dairy changes were also mainly due to decreased consumption but this was more likely to happen between ages 30–60 years. The main result is a remarkable stability in consumption of all of these foods, particularly after 40 years of age.

**Figure 3:**
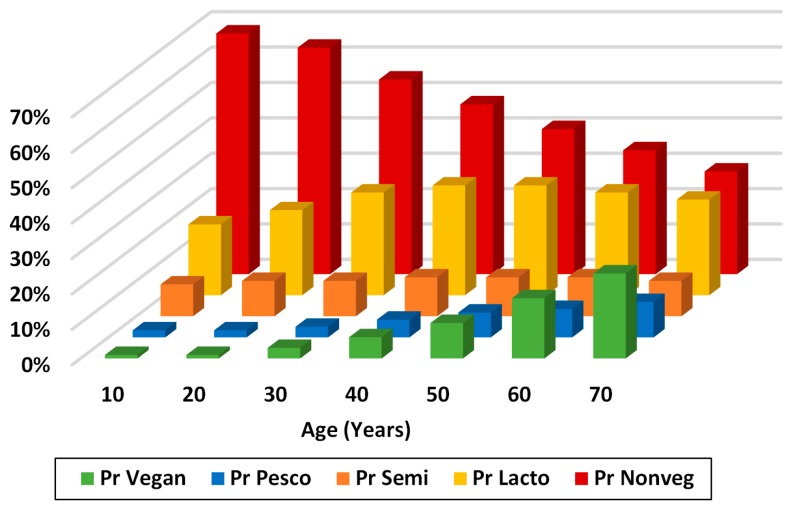
Proportions (Pr) in different dietary categories at different ages—longitudinal analysis in AHS-2 adjusted for birth cohort and other covariates. Reported here is the reference cohort who enrolled in AHS-2 in their 60s (Nominal multinomial analysis. pr non-veg: pr non vegetarians, pr lacto: lacto-ovo vegetarians, pr vegan: vegans, pr semi: semi vegetarians, and pr pesco: pesco vegetarians; *N* = 51,082). Other variables in the model are: diet age, race, gender, education, birth cohort.

**Distribution of dietary patterns and duration of adherence to current pattern.** The distribution of the dietary patterns by decade of life (standardized to birth cohort with current age 60) in longitudinal analyses is shown in Figure 3 (adjusted for birth cohort, also for differences in gender, race, and education). As subjects transition from their first to the last reported decade of life there was a clear tendency to move toward a more plant-based pattern, non-vegetarians to lacto-ovo- or pesco-vegetarians, and lacto-ovo vegetarians to vegans. The average proportion of non-vegetarians decreased from 68 to 29% with aging; lacto-ovo vegetarians increased from 20 to 31% during the first four decades and then decreased to 27% by the 7th decade; vegans, pesco vegetarians, and semi vegetarians increased from 1 to 24%, 2 to 10%, and 9 to 10%, respectively. Further details include that of all individuals who were lacto-ovo-vegetarians at age 40 years and who later switched, there was, as expected, a greater tendency to move toward a more vegetarian option. By the last decade, 66% of such switchers became vegans, 12% became pesco vegetarians, and fewer changed to eat more animal foods by becoming semi-vegetarians (12%) or non-vegetarian (10%).

**Figure 4:**
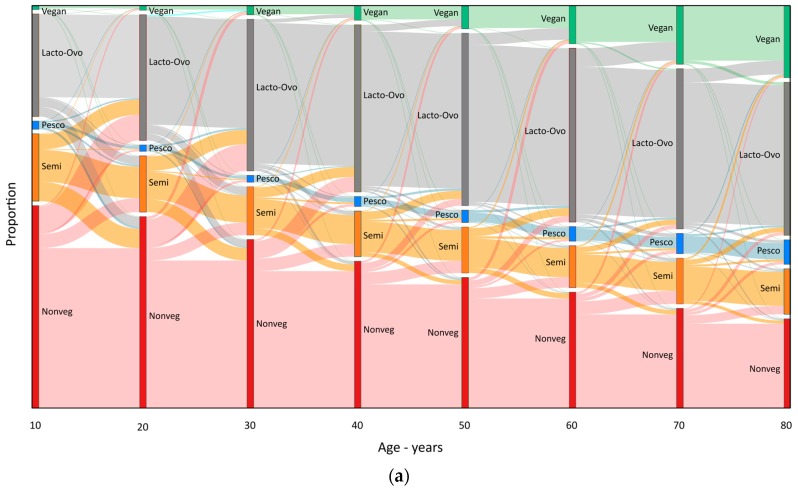

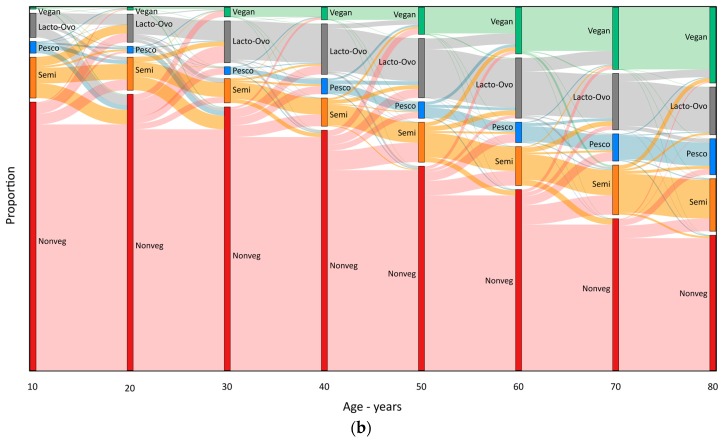
(**a**) Transition of vegetarian dietary patterns by decade for the non-Black 80+ years of age birth cohort (*n* = 4935). Vegan, Lacto-Ovo, Pesco, Semi, and Nonveg10, 20, 30, 40, 50, 60, 70 and 80 represent the proportion of individuals who were vegans, lacto-ovo vegetarians, pesco vegetarians, semi vegetarians and non-vegetarians at age 10 to 80, respectively. The width of transition lines indicate the proportions of the population involved; (**b**) Transition of vegetarian dietary patterns by decade for the Black 80+ years birth cohort (*n* = 357). Vegan, Lacto-Ovo, Pesco, Semi, and Non-veg 10, 20, 30, 40, 50, 60, 70 and 80 represent the proportion of individuals who were vegans, lacto-ovo vegetarians, pesco vegetarians, semi vegetarians and non-vegetarians at ages 10 to 80, respectively. The width of transition lines indicate the proportions of the population involved.

Figure 4a,b graphically illustrate the transition of dietary patterns over the lifetime among those who were very old (age ≥ 80 years) at HHF-3 (*n* = 5292), thus essentially capturing all their transitions. Figure 4a describes changes in non-Black subjects and 4b in Black subjects. Examination of separate figures for males and females (not shown) found almost identical patterns. The number of subjects making a particular transition is indicated by the thickness of the lines linking different patterns. Overall, movements toward more vegetarian patterns predominated (mainly non-vegetarian→lacto-ovo vegetarian, non-vegetarian→semi vegetarian, and semi vegetarian→lacto-ovo vegetarian). However, during their earlier decades these changes were almost counterbalanced by movements toward more non-vegetarian patterns (semi vegetarian→non-vegetarian, lacto-ovo vegetarian→non-vegetarian and lacto-ovo vegetarian→semi vegetarian), a trend that decreased markedly over time. Thus, at older ages, the most prominent diet patterns were the more vegetarian (15.5% vegan, 30.5% lacto-ovo vegetarian, 8.2% pesco vegetarian, but 12.0% semi vegetarian and 33.8% non-vegetarian). Moreover, the width of the lines representing the number of switchers of all sorts markedly decreased over time showing the much greater stability of diets at older ages. Overall mean (SD) reported durations of adherence to the current dietary pattern was 28 ± 24 years. Mean durations were 36 ± 25 years for non-vegetarians, 30 ± 23 years for lacto-ovo vegetarians, 11 ± 14 years for semi vegetarians, 14 ± 13 years for vegans, and 9 ± 11 years for pesco vegetarians.

**Characterization of the lifetime dietary pattern adherers and switchers.** Stability of dietary pattern was the most usual dietary characteristic of the overall study group with the majority of subjects remaining in the same pattern from one decade to the next (see Figure 2 and Figure 4). However, stability across the lifetime was less common (29%). A total of 71% of the 70+ year-old cohorts (they are those with a near lifetime experience to analyze) were switchers. Of the lifetime stable (LTS) adherers, 1.1% were LTS-vegans, 31.2% LTS-lacto-ovo vegetarians, 0.49% LTS-pesco vegetarians, 3.7% LTS-semi vegetarians, and 63.5% were LTS-non-vegetarians (see Table 1 and Table 2).

Table 1 shows the demographic and lifestyle correlates of these five lifetime stable dietary pattern cohorts among the 70+ year old participants. LTS-semi-vegetarians and LTS-vegans were the oldest and LTS-non-vegetarians the youngest group. Women were over-represented in the LTS-pesco vegetarian group (small numbers). LTS Black subjects showed a preference for a non-vegetarian diet and were much less represented in the LTS lacto-ovo vegetarian group, whereas LTS non-Blacks were mainly lacto-ovo vegetarians. LTS-non-vegetarians were much more likely to be overweight or obese and were less educated than other groups. They also showed a poorer lifestyle pattern in other ways, being prone to spending more time watching TV, and having tried alcoholic beverages more often in the last 12 months. The proportion of ever tobacco users was also higher among these individuals. The LTS non-vegetarians also tended to be newer Adventists with a lower average duration of church membership. Lifetime vegans were leaner and the most physically active, while lacto-ovo vegetarians were predominantly lifetime Adventists.

Among the 70+ year old Switchers, 6467 (60%) were “Converters”, 983 (9%) were “Reverters”, and 3384 (31%) were “Multiverters”. In Table 2 it can be seen that reverters (changing to eat more animal products) as compared to converters (changing to less animal product consumption) were generally more similar to LTS non-vegetarians than LTS vegetarians, with the exceptions that they were less likely to be Black, and had a tendency to be Adventists of longer duration.

## 4. Discussion

This is one of relatively few papers in the literature that explore patterns of eating across the lifetime. In this report, we show that the dietary habits of the AHS-2 cohort are dynamic with strong systematic trends in the consumption of animal products that tend to be eaten less with increasing age. Specifically, with aging Adventists tend to shift from less vegetarian toward more vegetarian dietary patterns. Despite this, on average the current dietary pattern had been stable for 28 ± 24 years as the rate of change was highest in the first 3–4 decades of life. The most stable patterns were the most common, specifically the lacto-ovo-vegetarian and non-vegetarian diets. The individuals described and characterized as lifetime stable, and switchers, comprise sub-cohorts whose disease experience can be compared in future studies. Some indices and analytic methods have been identified that helped us explore such changes in diet. Although these analyses are specific to American Adventists, it is possible that the trends and patterns we found also are occurring in other populations.

Since 1863, when the Seventh-day Adventist church was organized, members have been encouraged to adopt a healthful lifestyle to promote health, happiness, and enhanced spirituality [32]. Hence, Adventists support a “whole-person” model of health and well-being, which includes physical, mental, and spiritual dimensions [33]. Through their publications, church activities, social events, and institutions, Adventists promote a healthy lifestyle, including a vegetarian diet. These influences may stimulate the progressive changes during the lifespan towards the reduction of consumption of animal products. However recently there have been societal dietary trends in the same direction [34] influenced by publicity about the beneficial health effects, but also benefits to planetary ecology and animal welfare [35,36,37,38,39,40,41,42,43,44,45,46,47,48]. Older subjects may be particularly responsive to lifestyle recommendations, given the expectation of reduced risk of chronic disease.

As compared to lifetime vegetarians, the lifetime adherence to a non-vegetarian pattern in the Adventist cohort is more frequent among those with lower education, poorer lifestyle in other ways, higher BMI, and shorter time as a church member. This may suggest that it is particularly those Adventists who are more knowledgeable and have greater support and resources who are more likely to be vegetarian. Epidemiologic studies [49], some with Adventists [33,50,51,52], also show that church attendance is positively associated with healthy behaviors (i.e., exercise, non-smoking, low or absent meat and regular nut consumption), and with outcomes such as subsequent risk of incident coronary heart disease and all-cause mortality.

Switchers who were reverting to eat more animal products were in several respects like lifetime non-vegetarians. It is possible that they had previously struggled to maintain vegetarianism or perhaps had never been persuaded of its advantages. Characteristics of those converting to eat less animal food consumption include an overweighting of Blacks, being better educated and newer Adventists. This last suggests that many became adherers to the lifestyle recommendations upon joining the church.

No doubt a number of determinants interact in the initiation and maintenance of Adventist healthy behaviors, such as: values (religious beliefs, especially if instilled during childhood), perceived health consequences, and perceived social support, community norms and self-efficacy (i.e., skills) [53]. When a member is baptized in the Adventist church, typically in teenage years, he is encouraged to keep his body as healthy as possible. This may be why the highest rate of dietary change was in the younger decades. Yet this may also be a societal phenomenon as these are years of great change, leaving the parental home, marriage, and raising families.

The design of the questionnaire may miss dietary changes of very short duration (e.g., a 65 year old was non-vegetarian till 62 and then became vegetarian, but the questionnaire captured the most recent habits for such subjects only at age 60 years). The apparent long average duration of adherence to current patterns provides useful information in that it enables a more confident interpretation of previous reports from AHS-2 linking vegetarian dietary patterns to risk of disease and mortality [6,14,16,17,19,20,21,54] by indicating that our results would uncommonly have been confounded by recent switching to the current diet. It could be speculated however, that lifetime vegetarian patterns and distinct patterns of switchers described in the present study may have different effects on the risk of disorders affected by vegetarian status [12].

We and others have found associations between duration of vegetarian diet or low meat intake and a decrease in all-cause mortality raising the possibility that long term maintenance of these dietary patterns may be most effective in reducing all-cause death rates [55,56,57,58,59]. For instance, Key et al. (1999) combined data from 5 prospective studies containing many vegetarians [60]. They found that the lower mortality from ischemic heart disease among vegetarians was restricted to those who had followed their current diet for >5 years. Rosell, Appleby and Key (2006) in the EPIC-Oxford study also found the least weight gain over five years’ follow-up among those who had switched to a diet containing fewer animal foods [15]. Both duration of and changes between dietary patterns may be considered when addressing health outcomes.

Others who studied lifetime dietary habits [61,62,63,64,65,66,67], usually did so with a specific aim of estimating lifetime exposure to specific foods and/or nutrients such as intake of milk and/or calcium, or meats and polycyclic aromatic hydrocarbons and heterocyclic amines, or to assess the reliability of recalled intake of specific foods captured at different stages of life [26,62]. The stability or duration of adherence to a particular dietary habit was not formally quantified or assessed in these studies. To our knowledge this is the first study that provides detailed description of these attributes.

Possible weaknesses of this study reflect the limitations of the instrument used for recall of lifetime consumption of animal foods discussed elsewhere [26,27], this particularly relating to recall and other sources of dietary measurement error. However, we have demonstrated good reliability of recall of such data over a time lapse of more than 30 years [27]. Another probably minor issue is that the value estimated to represent frequency of consumption for the open-ended category 1+ per week is based on the more detailed frequency data obtained from the cohort in the baseline FFQ. This may have varied somewhat from birth cohort to birth cohort.

## 5. Conclusions

We conclude that among subjects of the Adventist Health Study-2 (AHS-2) cohort the main trends in lifetime consumption of animal products are towards the reduction of these foods and changes to more vegetarian patterns. Nevertheless, adherence to the current diet in this middle-aged and older population is typically measured in decades. The hypothesis that lifetime exposures to vegetarian patterns, and also particular patterns of dietary change, could better express the relationship between diet and diseases with long incubation periods should be tested in future work.

## Figures and Tables

**Table 1:** Distribution of non-dietary variables among Adventists aged 70+ years who were lifetime-stable adherents to particular dietary patterns *.

	LTS-Vegan	LTS-Lacto	LTS-Semi	LTS-Pesco	LTS-Non-veg	Switchers	*p*
*n*	52	1403	165	22	2856	10,834	
Age in years, mean (SD)	78.85 (5.22)	78.45 (5.40)	78.87 (5.30)	78.23 (5.07)	76.78 (4.93)	77.52 (5.20)	<0.001
Gender, %							<0.001
Male	36.5	36.3	43.6	27.3	42.5	38.3	
Female	63.5	63.7	56.4	72.7	57.5	61.7	
Race, %							<0.001
Non-black	86.5	98.9	94.5	95.5	82.6	91.0	
Black	13.5	1.1	5.5	4.5	17.4	9.0	
BMI in kg/m^2^, %							<0.001
Underweight (<18.5)	2.0	2.1	1.3	9.5	1.0	2.2	
Normal (18.5–24.9)	55.1	46.6	29.9	28.6	26.1	43.6	
Overweight (25–29.9)	34.7	37	49	33.3	43.6	36.6	
Obese (≥30)	8.2	14.3	19.7	28.6	29.3	17.7	
Education, %							<0.001
High school or less	26.9	12.4	28.5	31.8	34.5	23.6	
Some college	48.1	35.3	38.2	31.8	40.8	39.1	
College or higher	25	52.3	33.3	36.4	24.7	37.4	
Exercise, %							0.027
None	63.2	69.8	70.8	60.0	68.7	69.7	
1–20 min/week	10.5	15.3	11.8	6.7	13.3	13.9	
21–60 min/week	7.9	9.2	11.8	26.7	10.4	9.8	
61–150 min/week	7.9	3.6	2.8	0	5.3	4.6	
≥151 min/week	10.5	2.1	2.8	6.7	2.2	2	
TV watching, %							<0.001
None to <1 h/day	38.5	26.3	12.1	22.7	11.1	22.5	
1–2 h/day	48.1	52.8	54.5	54.5	44.3	49.4	
≥3 h/day	13.5	20.9	33.3	22.7	44.6	28	
Sleep, %							<0.001
<6 h/day	21.2	21.9	23	45.5	32.2	27.9	
7 h/day	42.3	36.4	31.5	22.7	31.6	34.8	
≥8 h/day	36.5	41.8	45.5	31.8	36.2	37.3	
Alcohol, past year, %							<0.001
No	100	99.2	95.2	100	91.2	97.5	
Yes	0	0.8	4.8	0	8.8	2.5	
Cigarette smoking, %							<0.001
Never	96.2	98.5	89.7	100	67.1	83.3	
Ever	3.8	1.5	10.3	0	32.9	16.7	
Years since baptism into Adventist church, mean (SD)	60.18 (13.73)	65.54 (6.37)	59.44 (14.41)	60.25 (14.89)	44.80 (20.52)	56.56 (14.75)	<0.001
Lifetime Adventists, %							<0.001
No	28.8	5.1	26.7	22.7	63.7	41.8	
Yes	71.2	94.9	73.3	77.3	36.3	58.2	

* Analyses of variance (ANOVAs) were used to check for differences in the means of continuous covariates and Chi-Square tests were used to check for differences in proportions among categorical covariates. SD is standard deviation.

**Table 2:** Distribution of non-dietary variables among Adventists aged 70+ years who were switchers (converters, multiverters, reverters) between dietary patterns *.

	Level	Converter	Multiverter	Reverter	*p*
*n*		6467	3384	983	
Age in years, mean (SD)		77.46 (5.19)	77.63 (5.26)	77.56 (5.10)	0.288
Gender, %	Male	40.1	35.5	36.1	<0.001
	Female	59.9	64.5	63.9	
Race, %	Non-black	90.6	91.1	93.5	0.011
	Black	9.4	8.9	6.5	
BMI in kg/m^2^, %	Underweight (<18.5)	2.6	1.9	0.8	<0.001
	Normal (18.5–24.9)	46.9	40.9	30.5	
	Overweight (25–29.9)	34.4	38.8	43.1	
	Obese (≥30)	16.1	18.3	25.5	
Education, %	High school or less	23.5	23.1	25.4	0.001
	Some college	37.8	40.6	41.8	
	College or higher	38.7	36.2	32.8	
Exercise, %	None	68.6	70.1	75.7	0.002
	1–20 min/week	14.3	13.5	13	
	21–60 min/week	10.1	9.8	7.6	
	61–150 min/week	4.7	4.8	2.6	
	≥151 min/week	2.3	1.9	1.1	
TV watching, %	None to <1 h/day	24.7	20.9	14.4	<0.001
	1–2 h/day	48.7	51	48.9	
	≥3 h/day	26.6	28.2	36.6	
Sleep, %	<6 h/day	26.9	29.2	29.6	0.001
	7 h/day	36.4	32.7	31.8	
	≥8 h/day	36.7	38.1	38.6	
Alcohol, past year, %	No	98.8	96.7	92	<0.001
	Yes	1.2	3.3	8	
Cigarette smoking, %	Never	83.5	83.2	82.4	0.678
	Ever	16.5	16.8	17.6	
Years since baptism into Adventist church, mean (SD)		55.71 (14.94)	57.73 (14.03)	58.12 (15.56)	<0.001
Lifetime Adventists, %	No	46.5	37.5	26.4	<0.001
	Yes	53.5	62.5	73.6	

* ANOVAs were used to check for differences in the means of continuous covariates and Chi-Square tests were used to check for differences in proportions among categorical covariates. SD is standard deviation.
